# Soil Microbial Communities Significantly Changed Along Stand Ages in Masson Pine (*Pinus massoniana* Lamb.) Plantation

**DOI:** 10.3390/plants14193004

**Published:** 2025-09-28

**Authors:** Weijun Fu, Bingyi Wang, Dunzhu Li, Yong Zhang

**Affiliations:** 1 State Key Laboratory of Subtropical Silviculture, Zhejiang A&F University, Hangzhou 311300, China; fuweijun@zafu.edu.cn; 2 College of Jiyang, Zhejiang A&F University, Zhuji 311800, China; lidunzhu@zafu.edu.cn; 3 Zhejiang Provincial Management Station of Public Welfare Forest and State-owned Forest Farms, Hangzhou 310020, China

**Keywords:** Masson pine (*Pinus massoniana* Lamb.) plantation, microbial community structures, soil nutrients, soil organic carbon

## Abstract

Soil microbial communities are important for nutrient cycling regulation in forest ecosystems. However, limited knowledge exists regarding the characteristics of these microbial communities in Masson pine (*Pinus massoniana* Lamb.) plantations of different stand ages. In this study, four planted Masson pine stands (8-year-old, 12-year-old, 22-year-old, and 38-year-old stands) and one natural broadleaved forest stand (as a control) with three replications, were selected in the Laoshan Forest Farm, Qiandao Lake Town, Zhejiang Province, China. Soil physicochemical properties were measured and their effects on soil microbial communities were studied. Amplicon-based high-throughput sequencing was employed to process raw sequence data for soil microbes. It is worth noting that significant differences (*p* < 0.05) in soil bacterial genera were observed among different stand age groups. Total nitrogen (TN), total phosphorus (TP), total potassium (TK), available potassium (AK), soil organic carbon (SOC), and soil bulk density (BD) were identified as the primary factors influencing bacterial community distribution (*p* < 0.05). Available nitrogen (AN), SOC, TN, and TK showed significant correlations with soil fungal communities (*p* < 0.05). These findings underscore the crucial role of soil physicochemical properties in shaping soil microbial community composition in Masson pine plantations.

## 1. Introduction

Soil microorganisms significantly contribute to organic matter decomposition, enhancing soil structure and fertility and participating in essential processes like soil formation, nutrient cycling, and sustainable plant development [1,2,3]. Therefore, the community structure of soil microorganisms serves as a vital indicator of terrestrial ecosystem dynamics such as biochemical cycling and material exchanges on Earth [4,5,6,7]. Meanwhile, vegetation plays a pivotal role as primary producers, providing essential nutrients to soil microorganisms. These microorganisms function as decomposers, converting organic matter into vital nutrients [8,9]. Therefore, comprehending the diversity and community composition of soil microorganisms is crucial for elucidating the intricate feedback mechanisms among forest tree species [10], microorganisms, and soil dynamics [11]. Studies have demonstrated that environmental factors such as soil nutrient contents [12], pH levels [13], and forest/plantation age [14] regulate the structure of soil microbial communities. Previous research has also confirmed that alterations in soil organic carbon (SOC) and total nitrogen (TN) levels can significantly impact the composition of soil bacterial communities [15]. Moreover, processes like mineralization and patchy nutrient utilization patterns executed by soil microorganisms substantially influence vegetation’s nutrient uptake capacity [16].

According to the 9th China Forest Resources Inventory (2014–2018), the national forest coverage rate was about 22.96% [17], corresponding to a total area of 220 million hectares, with an estimated national forest stock volume of 17.56 billion cubic meters [18]. Forest ecosystems serve a critical function in maintaining the homeostasis of global ecological equilibria, exerting regulatory influences across multiple biogeochemical and ecological processes [19], while plant/microbe interactions are indispensable for soil fertility and the functionality of forest ecosystems. Therefore, identifying the shifts in soil microbial communities holds significant ecological importance for understanding forest ecosystems [20]. However, limited research has been conducted on the variations in soil microbial diversity and community composition across different age stages of Masson pine (*Pinus massoniana* Lamb.) plantations (MPPs), particularly within subtropical coastal regions of China.

Previous studies have revealed that vegetation composition affects soil microbial activity in coniferous forests [21,22]. Moreover, metagenomic analysis has shown a progressive increase in forest-associated soil bacteria and fungi with advancing forest age [23,24]. Additionally, changes in soil enzyme activity resulting from the impact of increasing plantation age on microbial community structure can subsequently affect soil nutrient status [25,26]. However, large-scale afforestation efforts have led to the establishment of MPPs with varying ages [27], further influencing the overall soil microbial community structure within these ecosystems [28]. Therefore, accurately assessing variations in soil microbial diversity [29] in MPPs of different ages is crucial for comprehending their growth conditions and understanding the status of soil nutrients [30,31,32].

This study investigated the soil microbial community structure and physicochemical properties of MPPs across different age classes (8-year-old young forest, 12-year-old middle-aged forest, 22-year-old mature forest, and 38-year-old over-mature forest), with natural broadleaved forest (NBLF) soil samples serving as the control group. We hypothesized that (1) Forest stand age exerts significant influences on both the physicochemical properties of soil and the diversity of microbial community composition; (2) the effects of forest age on soil bacterial and fungal communities are non-unidirectional and exhibit complex regulatory patterns; and (3) the key driving factors governing soil bacterial and fungal communities differ substantially in their underlying mechanisms.

## 2. Results

### 2.1. Physical and Chemical Properties of the Soil

As depicted in Table 1, the soil pH value of MPPs across different forest ages was consistently acidic. Soil organic matter was significantly higher (*p* < 0.05) in both the mature (MPP (38-year-old)) and young forest (MPP (8-year-old)) stages compared to other stages. The contents of soil total nitrogen, phosphorus, and potassium were slightly higher in mature forests, although not statistically significant (*p* > 0.05).

### 2.2. Soil Microbial Community Structure Variation Along the Stand Ages

**Figure 1:**
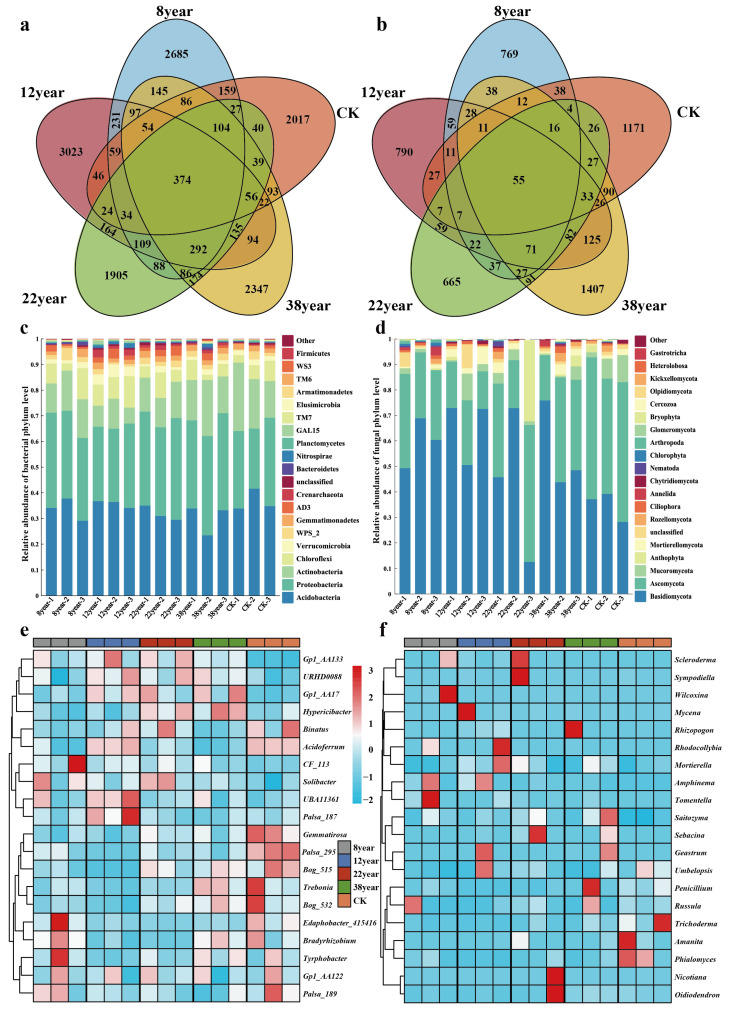
The OTU number of soil bacteria (**a**) and fungi (**b**) in forests of different ages based on Venn analysis. The relative proportion of the top twenty soil bacterial (**c**) and fungal (**d**) phyla in forests of different ages. The clustering heatmaps of the most important twenty soil bacteria (**e**) and fungi (**f**) genera in forests of different ages.

The Venn analysis of soil bacterial and fungal operational taxonomic units (OTUs) in the soils of MPPs with different stand ages revealed that bacteria and fungi shared 16,350 OTUs, including 11,603 bacterial OTUs and 4747 fungal OTUs (Figure 1a,b). Among these, 374 bacterial OTUs and 55 fungal OTUs were shared across all different stand ages, respectively. The identified bacterial OTUs spanned 37 phyla, 97 orders, and 171 genera, while the fungal OTUs encompassed 31 phyla, 72 orders, and 602 genera. The number of age-specific bacterial and fungal OTUs initially increased, then decreased, before increasing again as forest aged. In comparison, the number of these OTUs exceeded that observed in natural forests.

The top 20 dominant phyla of bacterial and fungal communities in soil across different stand ages are shown in Figure 1c,d. Among soil bacteria, Acidobacteria and Proteobacteria exhibited a higher relative abundance, ranging from 30.18% to 36.78% and 29.36% to 36.96%, respectively. While there was no significant change in the relative abundance of Acidobacteria or Proteobacteria with the increase of forest age (*p* > 0.05), Actinobacteria showed a significant increasing trend (*p* < 0.05, Table 2).

Among fungi, Basidiomycota and Ascomycota were identified as the dominant phyla, accounting for 34.85–65.35% and 19.44–51.91% of the relative abundance, respectively. As forest age increased, a decreasing trend was observed regarding the relative abundance of Ascomycota. Overall, the dominant phyla of both bacteria and fungi did not show significant changes while variations in their respective relative abundances were noted.

Further comparisons were conducted between dominant bacterial and fungal genera in the soils of Masson pine under different stand ages (Figure 1e,f). Variations in the distribution of dominant bacterial and fungal genera were observed in Masson pine soils with different forest ages. Regarding bacterial genera, *Palsa_187*, *UBA11361,* and *URHD0088* were predominantly enriched in the soil of 12-year-old forest, while *Edaphobacter_415416*, *Bradyrhizobium*, *Tyrphobacter,* and *CF_113* were enriched in the 8-year-old forest. Interestingly, the bacterial genera in the soils of 22-year-old and 38-year-old forests exhibited similar distribution, with *Gp1_AA17*, *Hypericibacter,* and *Binatus* being the main enriched genera. At the fungal genera level, *Wilcoxina* and *Tomentella* were significantly enriched in the soil of 8-year-old forest, while the soil of 12-year-old forest exhibited a higher abundance of enriched genera. In comparison, *Scleroderma* and *Sympodiella* were enriched in the soil of 22-year-old forest, whereas *Rhizopogon* and *Penicillium* were primarily enriched in the soil of 38-year-old forest.

### 2.3. Structural Diversity of Soil Microbial Community

The Kruskal–Wallis H-test indicated significant differences (*p* < 0.05) in the dominant bacterial phyla (Actinobacteria) among Masson pine of different forest ages (Table 2), whereas the dominant fungal phyla did not show significant differences (*p* > 0.05). Additionally, the bacterial α diversity of Masson pine was found to be significantly higher than that of natural broadleaved forests, as confirmed by a two-factor analysis of variance (*p* < 0.05, Table 3).

**Figure 2:**
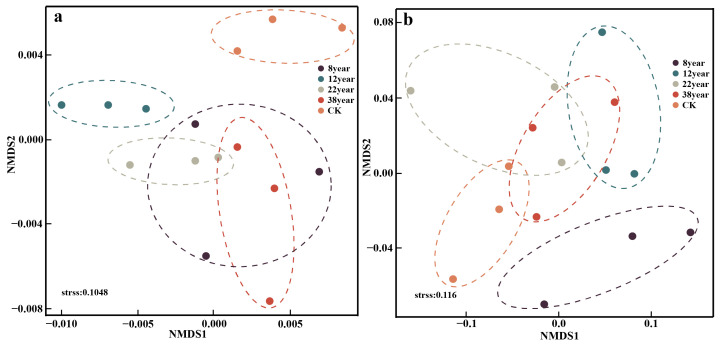
Non-metric multidimensional scaling (NMDS) analysis of soil bacterial communities (**a**) and fungal communities (**b**).

The β-diversity of soil bacterial and fungal communities was further investigated using non-metric multidimensional scaling (NMDS) to explore the variations across different forest ages (Figure 2). The NMDS analysis revealed that soil exhibited higher similarity within the same forest age, while more pronounced differences were observed in different forest ages. Figure 2a clearly demonstrated notable variations in bacterial community across different forest ages. Importantly, the analysis also revealed that the younger forests exhibited greater divergence in bacterial community structures. The divergence in bacterial community structure increased with forest ages. Similarly, the youngest subgroup (8-year-old forest) exhibits the greatest disparity in fungal community structure when compared to other subgroups.

### 2.4. The Associations Between Soil Microbial Communities and Soil Properties

**Figure 3:**
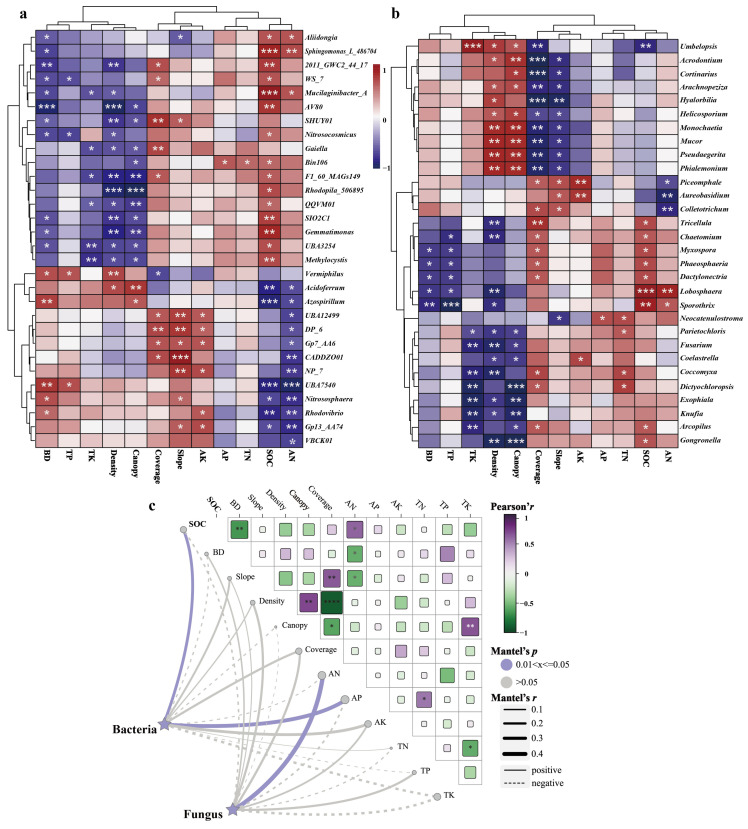
The correlation between soil bacteria genera and environmental factors (soil physicochemical properties, vegetation factors, and slope) (**a**) and soil fungus genera and environmental factors (**b**). Correlation heatmap for soil microbia and fungi with environmental factors based on Mantel test (**c**). *: stands for significant difference at *p* < 0.05. **: stands for significant difference at *p* < 0.01; ***: stands for significant difference at *p* < 0.001.

Soil physical and chemical parameters such as bulk density (BD), total nitrogen (TN), and soil organic matter (SOC) showed significant positive correlations with bacterial genera such as *Vermiphilus* and *Acidoferrum* (Figure 3), but the relationship between available phosphorus (AP), total nitrogen (TN), SOC, available nitrogen (AN), and bacterial genera exhibited an inverse pattern compared to the previous findings. Conversely, the correlations between BD, total phosphorus (TP), total potassium (TK), Density, Coverage, Slope, available potassium (AK), AP, TN, SOC, AN, and fungal genera generally demonstrated contrasting tendencies, as depicted in Figure 3b. The relationships between BD, TP, TK, Density, Canopy, and *Tricellula*, *Chaetomium Myxospora* and other fungal genera exhibited significantly negative correlations. Overall, the associations between bacterial and fungal genera and soil physicochemical properties demonstrated distinct trends.

As depicted in Table 4 and Figure 3c, a significant correlation (*p* < 0.05) was observed between SOC and bacteria, as well as AP and bacteria. Additionally, a significant correlation (*p* < 0.05) was found between AN and the soil fungal community. Overall, SOC and available nutrients play the most prominent role in shaping soil bacterial and fungal communities.

**Figure 4:**
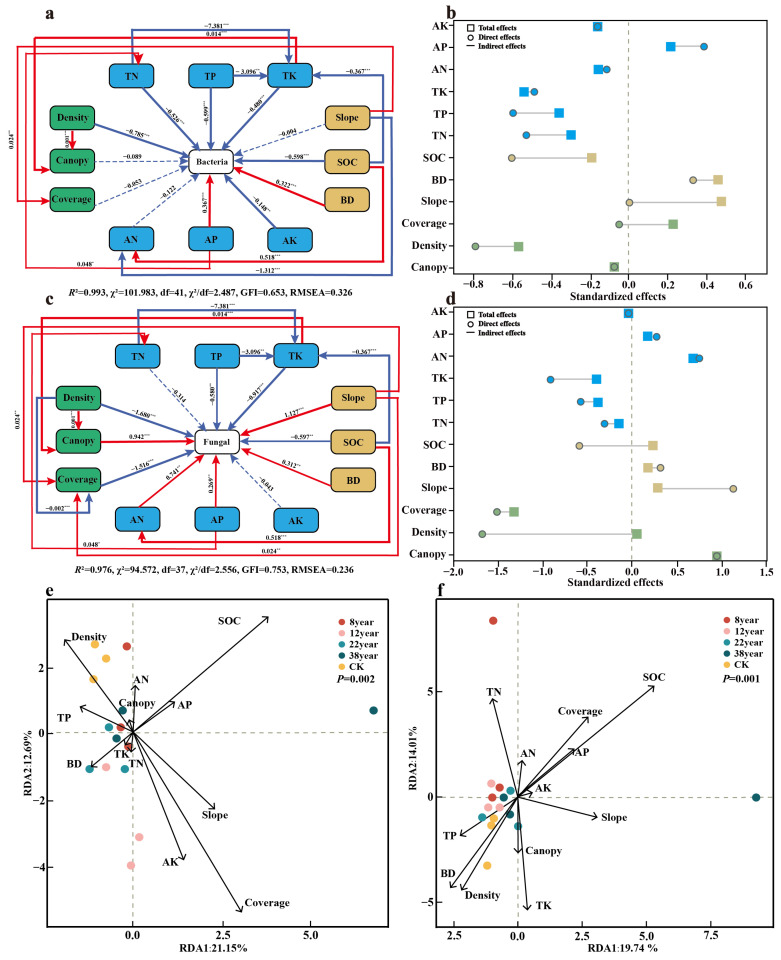
Structural equation model diagrams: (**a**) soil bacteria of Masson pine plantations and (**c**) soil fungi of Masson pine plantations. Figure (**b**,**d**) depict the standard effect diagrams within the structural equation model diagrams. (**e**) RDA of soil bacterial communities and (**f**) RDA of soil fungal communities. * *p* < 0.05; ** *p* ≤ 0.01; *** p ≤ 0.001.

The structural equation modeling was employed to reveal the influences of environmental factors (soil physicochemical properties, vegetation factors, and slope in this study) on soil microbial community structure, with results illustrated in Figure 4a–d. As depicted in Figure 4a, TN, TP, TK, AK, SOC, and BD exhibited significantly negative effects on the bacterial community structure (*p* < 0.001). Figure 4b indicates the direct effects on soil bacteria, ranked as follows: Density > SOC > TP > TN > TK > AP > BD > AK > AN > Canopy > Coverage > Slope. According to Figure 4c, TK, AK, SOC had highly significant negative effects on fungi community (*p* < 0.001), whereas capacity, Slope, AP, AN, and Canopy demonstrated highly significant positive impacts (*p* < 0.001). Figure 4d outlines the direct effects of each factor on soil fungi, ranked as follows: Density > Coverage > Slope > Canopy > TK > SOC > AN > TP > TN > BD > AP > AK.

The RDA1 and RDA2 collectively accounted for 33.84% and 33.75% of the total variation in bacterial and fungal community structures, respectively. For bacterial communities, SOC and vegetation cover were identified as the primary environmental factors influencing their distribution (*p* < 0.05). Similarly, SOC, TN, and TK emerged as the main environmental factors influencing the distribution of fungal communities (*p* < 0.05). Bacterial communities in natural forests were positively correlated with Density, TP, BD, TK, TN, and AN and negatively correlated with SOC and AP. Soil fungal communities across different forest ages demonstrated positive associations with TP, BD, Density, Canopy, and TK and negative associations with AN, SOC, AP, AK, Slope, and Coverage.

## 3. Discussion

### 3.1. Soil Microbial Community Structure Along the Stand Ages

This study aimed to characterize the variations in soil microbial communities and edaphic properties across different stand age classes of MPPs, focusing on dominant taxonomic groups, diversity indices, and key drivers influencing bacterial and fungal assemblages. Forest ecosystems serve as critical niches for soil microbial proliferation [33], which are crucial for maintaining soil quality, fertility, and vegetation health [5]. Prior research has established that the diversity of soil bacterial and fungal communities directly modulates the stability and functional processes of soil ecosystems [34]. The number of specific OTUs for both bacteria and fungi initially increased with forest ages, followed by a decrease. Overall, the number of OTUs was significantly higher than that observed in natural broadleaved forests. These findings confirm that stand age strongly influences the community structure of soil microorganisms while highlighting the fundamental role played by soil in driving changes within these microorganisms [22], which are consistent with our research findings. In addition, the growth of soil microorganisms is significantly influenced by soil pH [35]. The soil environment in Masson pine forests provides favorable conditions for bacterial growth [36], which contributes to the aforementioned findings. Moreover, compared to over-mature forests, middle-aged Masson pine forests exhibit higher diversity and abundance of bacteria and fungi. This could be attributed to the substantial impact of tree age on the interaction between vegetation and soil microorganisms. As forest age increases, Masson pines require more nutrients, thereby affecting the growth of soil microorganisms and ultimately exerting a negative influence on the microbial community in the soil [37]. Notably, both bacterial and fungal diversity and abundance in MPPs exceeded those documented in natural forest systems [38,39]. This discrepancy may be linked to the age structure of natural forests in the study region, which have remained unmanaged since establishment, leading to a generalized over-mature state. Such long-term lack of anthropogenic intervention is presumably a major contributor to reduced soil microbial diversity and abundance in natural forests [40,41].

The composition and structural complexity of soil microbial communities have been demonstrated to indirectly impact soil quality, thereby impeding the growth of forest vegetation [1]. In Masson pine forests of varying ages, *Acidobacteria* and *Proteobacteria* are identified as the dominant phyla among soil bacteria, while *Ascomycota* represents the dominant phylum among fungi. It is postulated that these dominant phyla exhibit enhanced adaptability within Masson pine forests, given the faster growth rate observed in bacterial communities under nutrient-rich conditions [42]. Consequently, Acidobacteria also emerges as a predominant phylum in Chinese fir and native pine forests of different ages [43]. Furthermore, these dominant phyla likely play a pivotal role as primary decomposers in complex organic matter degradation within soils, thus establishing their prominence within Masson pine forests [44]. The results of cluster analysis and RDA analysis demonstrate significant changes in bacterial composition with forest age in Masson pine forests, exhibiting higher bacterial diversity within the genus compared to natural forests. This disparity can be attributed to the increased abundance of nutrient-rich and eutrophic bacteria and fungi in the soil environment of resource-rich plantation forests [29], leading to variations in soil microbial community composition. in resource-rich plantation environments, which consequently lead to alterations in soil microbial community composition. Forest succession processes are known to influence plant community structure and organic carbon levels, thereby impacting microbial community activity [45]. Further research is essential for understanding the complex interactions between forest types and soil microbial activity.

The composition of soil microbial communities profoundly influences their dynamics and ecological functions [7]. The NMDS analysis revealed that bacteria and fungi in soils of the same forest age exhibited high similarity but significantly differed from those present in natural forests. Notably, bacterial communities displayed more pronounced variations among the different ages of Masson pine forests, while fungal communities showed relatively stable patterns across forest age. These findings suggest that tree species and age may play a crucial role as they create distinct internal environments, making conditions within Masson pine forest ecosystems more favorable for bacterial growth [46,47].

### 3.2. Factors Influencing the Dominant Flora and Diversity of Microbial Community Structure in Soil

Soil microorganisms play a pivotal role in soil ecosystems and exhibit high sensitivity to environmental fluctuations. Research findings have demonstrated that TN, TP, TK, AK, SOC, and Density exert a significantly negative influence on bacterial community indicators (*p* < 0.001), with soil organic carbon and vegetation coverage emerging as the primary environmental factors influencing bacterial community distribution (*p* < 0.05). These outcomes can be attributed to the substantial correlation between bacterial diversity and soil properties, particularly carbon and nitrogen elements [48]. The interplay of nitrogen (N) and carbon (C) elements synergistically affects bacteria’s assimilation of C while promoting N availability. Moreover, soil pH regulates changes in bacterial communities by modulating the living environment of soil bacteria through its impact on the efficacy of N and C within the soil matrix [49]. The above results occurred because bacteria and fungi are adapted to different environmental conditions, and the contents of C, N, and P in soil indirectly influence microbial growth.

The results of this study demonstrate a significant correlation (*p* < 0.05) between AN, SOC, TN, TK, and soil fungal community, supporting our hypothesis that the main influencing factors for bacteria and fungi are not entirely identical. Soil total nitrogen content is a crucial determinant of soil fungal abundance and diversity due to their wider pH tolerance range [50] and ability to acquire C in more diverse forms [24,32], making them more susceptible to higher N content [50]. Overall, soil physicochemical properties and environmental factors exert substantial impacts on the microbial community composition in soils across different forest ages [51]. Soil exhibits strong spatiotemporal variability in biological, physical, and chemical properties, creating a diverse and complex environment for microorganisms. The joint effects of vegetation density, terrain, and afforestation duration lead to variations in soil properties across different forest ages. As soil properties at different layers serve as primary drivers of changes in microbial community structure, the compositions of soil bacterial and fungal communities are consequently significantly influenced by soil nutrient contents. This knowledge motivates us to further investigate the mechanisms underlying interactions among soil/vegetation/microorganisms towards enhancing forest ecosystem functionality and contributing towards achieving carbon neutrality.

## 4. Materials and Methods

### 4.1. The Study Area and Sampling

The study area was situated in the Laoshan Forestry Field (29°33′30″ N, 119°02′55″ E) of Qiandao Lake, Hangzhou City, China. The area falls within the subtropical monsoon zone, characterized by a substantial annual precipitation of 1430 mm. The mean annual temperature was 17.1 °C, accompanied by an average of 1951 h of sunshine per year. The approximate average elevation is 150 m above sea level. The original vegetation was represented by typically subtropical evergreen broadleaved forest (*Cyclobalanopsis glauca* and *Castanopsis sclerophylla* species), with MPPs as the main forest communities since 1980s.

A “space-for-time substitution” method was applied to study the soil microbial community in MPPs with different stand ages. To minimize variation among different Masson pine stands, plantations with consistent soil type, slope incline, and topography were selected. The selected MPPs were planted in 1986, 2002, 2012, and 2016 after clearly cutting the original broadleaved forest. The stands consisted of a juvenile forest (8 years old, since 2016), a middle-aged forest (12 years old, since 2012), a mature forest (22 years old, since 2002), and an over-mature forest (38 years old, since 1986). Moreover, a nearby natural broadleaved forest (NBLF) with dominating *Cyclobalanopsis glauca* and *Castanopsis sclerophylla* was selected as the control. After a rigorous evaluation of the sites, three replicated plots (20 m × 20 m per each) for each age class of MPP and NBLF were randomly established, resulting in a total of 15 plots. A comprehensive survey was conducted to assess the vegetation density of Masson pine forests with a diameter at breast height (DBH) ≥ 3 cm, by measuring parameters such as DBH, tree height, and crown width on a tree-by-tree basis within each plot. Soil samples from a depth of 0–10 cm were collected. Soil samples weighing 500 g each, free from gravel, were transported to the laboratory. The main characteristics of the studied sites are summarized in Table 5.

### 4.2. Soil Properties Analysis

Soil physicochemical properties were determined according to the standard methods of Agricultural Chemistry Committee of China [52]. Soil organic matter was quantified using the potassium dichromate externally heated sulfuric acid oxidation method. Bulk density was measured by cutting ring method. The TN and TP were determined by semi-micro Kjeldahl method and alkali melt/molybdenum/antimony resistance colorimetric method, respectively. Alkali melt-flame spectrophotometry was used to assess TK. The available nitrogen (AN) was determined by alkaline hydrolysis diffusion method. Available phosphorus (AP) was extracted by sodium bicarbonate and then determined by molybdenum/selenium/antimony colorimetric method. Available potassium (AK) was extracted by ammonium acetate and determined by flame photometry method. Soil pH was measured potentiometrically with a water/soil ratio of 2.5:1.

### 4.3. Gene Sequencing Analysis

The soil DNA was extracted using the cetyltrimethyl ammonium bromide standard method [53]. The purity and concentration of DNA were then assessed by 1% agarose gel electrophoresis. To determine the purity and concentration of DNA, PCR amplification targeting the V3-V4 region of the bacterial 16S rRNA gene was performed with primers 341F (5′-CCTAYGGGRBGCASCAG-3′) and 806R (5′-GGACTACNNGGGTATCTAAT-3′) For PCR amplification of fungal rDNA ITS1 region, primers ITS1F (5′-CTTGGTCATTTAGAGGAAGTAA-3′) and ITS1R (5′-GCTGCGTTCTTCATCGATGC-3′) were used. Aliquots were pooled based on PCR product concentration and subsequently purified. Library preparation was carried out using Illumina TruSeq^®^ DNA PCR-Free Sample Preparation Kit. Sequencing was conducted on the NovaSeq 6000 PE250 platform [53].

The raw sequences were processed using the QIIME2 software package. Initially, sequences were trimmed to a length of 200 bp using cutadapt (v1.10), Pear (v1.9.4), and Prinseq-liteV 0.20.4 [54]. Primer sequences were eliminated using cutadapt, followed by removal of tail region sequences with slightly lower quality values utilizing Prinseq-lite V0.20.4. Subsequently, paired-end reads were merged through Pear (v1.9.4) [55]. After eliminating single and chimeric sequences, high-quality sequences exhibiting ≥97% similarity were clustered into operational taxonomic units (OTUs) using Usearch (v5.2).

### 4.4. Statistical Analysis

Analysis of variance (ANOVA) was applied to investigate the impact of forest ages on soil physicochemical properties, the abundance and diversity of soil microbial communities. Prior to ANOVA analysis, the normality and homogeneity of variances were carried out in the data, with logarithmic transformations applied if necessary to ensure adherence to a normal distribution. When significance was observed based on the F statistic, mean comparisons were performed using Tukey’s test.

Statistical analyses were conducted using IBM SPSS Statistics 19.0 software, while R (ver. 3.6.1) was used to plot relative abundance, heat map, Venn analysis, redundancy analysis (RDA), and correlation heat map for analyzing the composition of soil microbial communities. To assess differences in microbial community composition among sample groups, a permutation-based multivariate analysis of variance with permutational multivariate analysis (PERMANOVA) was performed after applying Hellinger transformation to the OTU table. These results were further employed to analyze the structure of soil microbial community composition. QIIME2 software was used to assess α-diversity (Ace, Chao1, Pielou, Shannon index) and β-diversity of soil fungal communities, further examine the diversity and differences among soil microorganisms [56]. Additionally, structural equation modeling was conducted to reveal the correlation between soil microbial community structure and physicochemical properties.

## 5. Conclusions

This study explores the disparities in soil microbial community structural characteristics and soil properties among Masson pine forests of varying ages. Our findings reveal that distinct bacterial and fungal taxa exhibit differential roles across different forest ages, with the highest soil microbial abundance observed in the mature stage of Masson pine forests. Furthermore, significant differences (*p* < 0.05) in soil bacterial genera among forests of different ages, with the most abundant species of dominant soil bacterial genera found in the middle-aged forest (12 years). Additionally, bacterial and fungal diversity and abundance were higher in young and middle-aged Masson pine forests compared to over-mature forest. Moreover, both soil bacterial and fungal communities showed significant correlations (*p* < 0.05) with soil AP and SOC contents. Therefore, it is imperative to implement further soil fertility management and tree age control measures in order to effectively enhance the soil microbial activity within the Masson pine forest ecosystem, thereby making a significant contribution towards augmenting its carbon sequestration capacity.

## Figures and Tables

**Table 1:** Soil properties of forests at different ages.

Stand Types	pH Value	Organic Matter (g·kg^−1^)	Total Nitrogen (g·kg^−1^)	Total Phosphorus (g·kg^−1^)	Total Potassium (g·kg^−1^)
MPP(8-year-old)	4.96 ± 0.08 ^b^	56.97 ± 0.86 ^a^	1.31 ± 0.09 ^a^	2.23 ± 1.08 ^a^	23.74 ± 1.29 ^b^
MPP(12-year-old)	5.11 ± 0.09 ^a^	26.16 ± 3.62 ^b^	2.17 ± 0.14 ^a^	1.96 ± 0.29 ^b^	36.06 ± 5.71 ^a^
MPP(22-year-old)	4.79 ± 0.08 ^c^	34.26 ± 3.06 ^b^	1.33 ± 0.55 ^a^	1.89 ± 0.20 ^b^	34.46 ± 1.18 ^a^
MPP(38-year-old)	4.93 ± 0.13 ^b^	63.88 ± 17.11 ^a^	1.84 ± 0.79 ^a^	2.26 ± 0.53 ^ab^	36.57 ± 5.14 ^a^
NBLF	4.78 ± 0.08 ^c^	56.48 ± 19.12 ^a^	1.47 ± 0.21 ^a^	2.33 ± 0.77 ^a^	35.14 ± 2.13 ^a^

Mean ± SD, n = 3. Different lowercase letters indicate significant differences between forest stands of different ages at a significance level of (*p* < 0.05). MPP: Masson pine plantation; NBLF: natural broadleaved forest.

**Table 2:** The dominant phyla of soil microorganisms.

		H-Value	*p*-Value
Bacteria	Acidobacteria	4.800	0.308
	Proteobacteria	8.900	0.063
	Actinobacteria	9.500	0.049 *
Fungi	Ascomycota	8.800	0.066
	Basidiomycota	5.689	0.224
	Mucoromycota	4.800	0.308

* indicates significant differences (*p* < 0.05).

**Table 3:** Soil microbial community and α-diversity indexes.

Microbial Category	Stand Types	Chao1 Index	Shannon Index	Simpson Index
Bacteria	MPP(8-year-old)	2137.48 ± 212.46 ^ab^	9.80 ± 0.29 ^a^	0.997 ± 0.0008 ^a^
	MPP(12-year-old)	2254.65 ± 91.45 ^a^	10.00 ± 0.10 ^a^	0.998 ± 0.0002 ^a^
	MPP(22-year-old)	1748.06 ± 335.69 ^ab^	9.66 ± 0.20 ^ab^	0.998 ± 0.0003 ^a^
	MPP(38-year-old)	1836.43 ± 623.81 ^ab^	9.65 ± 0.42 ^ab^	0.997 ± 0.0006 ^a^
	NBLF	1457.00 ± 451.92 ^b^	9.11 ± 0.36 ^b^	0.996 ± 0.0008 ^b^
Fungi	MPP(8-year-old)	476.00 ± 74.36 ^b^	4.91 ± 0.53 ^a^	0.89 ± 0.05 ^a^
	MPP(12-year-old)	598.67 ± 86.00 ^b^	5.84 ± 0.35 ^a^	0.95 ± 0.02 ^a^
	MPP(22-year-old)	513.33 ± 66.88 ^b^	5.09 ± 0.95 ^a^	0.90 ± 0.05 ^a^
	MPP(38-year-old)	841.33 ± 244.39 ^a^	5.80 ± 0.88 ^a^	0.92 ± 0.03 ^a^
	NBLF	625.00 ± 65.80 ^ab^	5.56 ± 1.02 ^a^	0.92 ± 0.05 ^a^

The presence of distinct lowercase letters indicates statistically significant differences in α diversity index among different forest ages (*p* < 0.05). MPP: Masson Pine plantation; NBLF: natural broadleaved forest.

**Table 4:** Correlation results between soil microbial community structure and environmental factors.

Environmental Properties	Bacterial Community Structure		Fungal Community Structure	
	*r*	*p*	*r*	*p*
SOC	0.266	0.028 *	−0.105	0.789
BD	−0.067	0.726	0.107	0.155
Slope	0.157	0.068	0.043	0.319
Density	0.069	0.33	0.190	0.145
Canopy	−0.059	0.593	−0.012	0.446
Coverage	0.185	0.153	0.192	0.133
AN	−0.051	0.571	0.435	0.025 *
AP	0.379	0.015 *	−0.129	0.835
AK	0.258	0.051	0.140	0.126
TN	0.000	0.477	−0.073	0.601
TP	−0.021	0.522	0.189	0.115
TK	−0.207	0.877	−0.213	0.982

The *r* value in correlation analysis represents the strength of the correlation, while the *p* value indicates its statistical significance (*p* < 0.05). * indicates significant differences (*p* < 0.05).

**Table 5:** Main site conditions (altitude, slope aspect, mean DBH, mean tree height and stand density) of the MPP and NBLF.

Stands	Altitude (m)	Slope Aspect	Mean Tree DBH (cm)	Mean Tree Height (m)	Stand Density (Plant/ha)
MPP(8-year-old)	146	Northwest	13.3	12.6	1800
MPP(8-year-old)	145	North	14.8	13.5	1600
MPP(8-year-old)	141	North	14.3	13.6	1700
MPP(12-year-old)	139	Southeast	14.5	14.8	2400
MPP(12-year-old)	158	Southeast	12.0	15.6	2500
MPP(12-year-old)	148	Southeast	13.5	9.8	2200
MPP(22-year-old)	148	Southwest	19.2	30.9	2300
MPP(22-year-old)	141	Northwest	25.2	23.9	2000
MPP(22-year-old)	129	Northwest	29.9	25.4	1900
MPP(38-year-old)	139	Southeast	33.9	29.0	1800
MPP(38-year-old)	144	Northwest	26.5	28.6	1900
MPP(38-year-old)	150	Northeast	26.1	23.8	1800
NBLF	126	North	14.6	21.1	2400
NBLF	128	North	26.6	30.4	2000
NBLF	131	North	16.4	22.0	1600

MPP: Masson pine plantation; NBLF: natural broadleaved forest. DBH: Diameter at breast height.

## Data Availability

The data presented in this study are available on request from the corresponding author.
